# Attention to Diversity in Compulsory Secondary Education

**DOI:** 10.3390/ejihpe10040082

**Published:** 2020-12-20

**Authors:** Elia Vázquez Varela, Iago Portela Pino, Víctor Domínguez Rodríguez

**Affiliations:** 1 Ministry of Culture, Education and Universities, 28004 Madrid, Spain; eliav@edu.xunta.es; 2 Department of Health Sciences, University Isabel I, 09003 Burgos, Spain; iago.portela@ui1.es; 3 University of Vigo, 36310 Vigo, Spain

**Keywords:** attention to diversity, ordinary and extraordinary measures, compulsory secondary education, quantitative research.

## Abstract

The attention to pupil diversity is still considered one of the main issues to solve in the current educational system. The objective of the study was to establish and analyze the differences between the knowledge and the use of ordinary and extraordinary measures in the attention to diversity from the point of view of compulsory secondary education teachers. A descriptive study was performed with a quantitative methodology, making use of a 452-teacher sample (Mean: 47, Standard Deviation: 8.42) using a survey. The results show a better understanding and frequency of use of the ordinary measures compared to the extraordinary ones. Results also maintain that among the most used we found the adequacy of the didactic planning as well as the support of the therapeutic pedagogy and language and auditory specialist. Moreover, the analysis reveals that the CMOEAD (ordinary and extraordinary measures for the attention to diversity) survey possesses psychometric properties which support its use in further studies. In conclusion, a better knowledge and use of ordinary and extraordinary measures in attention to diversity enables a forward leap in the quality and equity for pupils.

## 1. Introduction

The new demand which schools are facing regarding the attention to an increasing diversity of pupils is among of the reasons to incorporate new responsibilities to teacher tasks. Pupils with specific educational needs require fair and non-discriminatory treatment with maximum quality, within ordinary customary and adapted education for the characteristics and needs of all students [1,2,3]. As such, educative attention to diversity is conceived as a set of ordinary and extraordinary measures aimed to adapt the educative response to different characteristics, potentials, and rhythms and learning styles, as well as motivations, interests, and social and cultural situations of all pupils [4].

Consequently, attention to diversity is constituted in the set of educational actions aimed at responding to the different capacities; rhythms and learning styles; motivations and interests; and social, cultural, linguistic, and health situations of the students. It is a concept that must encompass all the students of the centers and that requires that all the teachers design actions aimed at adapting to the needs of each student, with all the resources of the center, including the organizational resources. However, although greater knowledge, training, attitude, reflection, involvement, coordination, work, and acceptance of diversity help the participation of all students, it is not enough to attend to diversity in an inclusive way.

In this context, schools as educational institutions are the reflection of diversity, which produces changes and improvements of a socio-cultural nature and rethinks the educational response as a way to address individual differences, which allow each of the boys, girls, and young people to advance and progress in the school system, regardless of their characteristics and the specific educational needs they present. Thus, in the secondary stage, the measures of attention to diversity must respond to the specific needs of the students, without impeding students’ ability to achieve the objectives and basic competencies of the stage, as well as obtain the degree of the same.

In fact, in the Compulsory Secondary Education (CSE) field, ordinary measures for the attention to diversity are all those which facilitate the adaptation of the prescribed curriculum, without significant alteration to the objectives, contents, evaluation criteria, to the socio-cultural context of the educational centers and pupils’ characteristics. Its aim is to give answers to different curricular knowledge, motivations, interests, social relations, strategies, styles, and learning paces, aiming to facilitate the achievement of the objectives and competencies established in the various educational processes. It is important to point out that these ordinary measures tend specially to the organization, in curricular settings and intervention, and in no case do they suppose a separation of the pupils from their group/class aspects [5]. Likewise, extraordinary measures for the attention to diversity are understood to be the ones aimed to give answer to the educational needs of pupils with specific educational needs, for which may be required significant modifications of the customary curriculum, and also suppose essential changes within the organizational field, as well as elements for access to the curriculum or educational modalities. Those measures that essentially revolve around curricular flexibility, grouping of pupils, and diversification in time and space management, can usually distance the pupil form the group/class [6,7].

In the majority of the research previously carried out, recognition of diversity is assumed as a precept which must take up the educative proposal that guarantees the equality of opportunities and the success of the pupils [8,9,10]. Its evolution, more focused on theoretical than experiential considerations, has surpassed a reductive view centered on pupils with more problems or lacks, for an education based on diversity; that is to say, it follows an inclusive hypothetical procedure, based on education for everyone [11,12,13].

As such, the attention to pupil diversity has become a main topic of increasing importance to educational research—going from a context focused on disability typologies and people with deficiencies to the study of the teacher’s own self-reality, teaching, and learning processes—that demands a new research model, in coherence with the new way of thinking of special education. Currently, the progress in the attention to diversity is centered on the improvement of the school as a whole [14], emphasizing as an emergent line of research conducted to foster inclusive policies; educational leadership; design and curriculum development; the promotion of a collaborative culture in the interculturality sphere; coexistence in educational centers; and dropout and educational exclusion, disability, university, work activity, and school-to-work relationships in inclusive contexts [15].

In educational centers, the idea that all students are different predominates, presenting educational characteristics that are shared by the majority and others that are their own and individual. From this perspective, educational attention to diversity includes all the actions that are carried out in order to prevent the differences that identify us as human beings from becoming unequal. It is adapting or adjusting the educational response to the characteristics of the learner. As a consequence, the measures towards attention to diversity are of enormous importance to adjust the pupils’ teaching-learning process, as its lack of judgment is related with an increased risk of school failure or dropout [3]. For that reason, it is fundamental that in the educative field as well as with research, adequate evaluation instruments be available for such measures [7,16]. 

Under these premises, all the attention-to-diversity measures (ordinary or extraordinary) have been collected for the CSE and are presented in the organic law for the improvement of educational quality [17], with the intention to provide a new coordinate to the educative response of a mainly heterogeneous pupil population. Due to the fact that, although the competent institutions offer a series of ordinary and extraordinary measures to improve the attention to diversity of students in secondary school, no study has concerned itself with finding their effectiveness.

This study establishes and analyzes the differences between knowledge and the use of ordinary and extraordinary measures in the attention to diversity from the point of view of the secondary education teachers of the Autonomous Community of Galicia (Spain). We undertook a descriptive and inferential study in which were set the following objectives and hypotheses:

- To assess the psychometric properties of the survey on the ordinary and extraordinary measures of attention to diversity in Secondary School Education (CMOEAD). (H_1_: It is inferred that the CMOEAD questionnaire presents adequate psychometric properties for the evaluation of measures of attention to diversity.)
- To check the knowledge and use of attention-to-diversity measures in Secondary Education Schools. (H_2_: It is predicted that there are discrepancies between knowledge and use in measures of attention to diverse students.)
- To analyze whether the demographic variables predict significant differences in the use of those measures. (H_3_: Socio-demographic variables are predicted to determine significant differences in the use of measures for the care of diverse students.)

## 2. Materials and Methods 

### 2.1. Participants

The random probabilistic sample was formed by 452 teachers from the Autonomous Community of Galicia (Spain). The mean age in the sample was 47.08 years (SD: 8.42). The female gender was predominant (62.6%) over males (37.4%), due to a predominantly female teaching population. Regarding their work career, 72.6% had more than 18 years of experience, 21.7% had between 12 and 18 years of experience, and 5.8% had less than 12 years of experience. Most of them had a bachelor’s degree (78.8%), fewer had a general degree (16.6%), and even fewer had a doctorate degree (4.6%). Regarding their origin, 57.1% came from low-population provinces (Lugo and Ourense) and 42.9% from provinces with a higher population (A Coruña and Pontevedra).

### 2.2. Research Instrument

A survey with two distinct parts was used to collect information. The first part, using “ad hoc”, included variable demographics: gender, age, work career, academic degree, and province where they worked. The second part compiled the attention-to-diversity measures for secondary education established in the organic law for the improvement of educational quality [17]. In this second part, the scale was made up of 21 items with two response options. In the first option, the knowledge of ordinary and extraordinary measures in the care of diverse students was evaluated (dichotomous scale: yes/no), and in the second option, the frequency of use of these measures in educational centers was evaluated (Likert scale: never, rarely, sometimes, frequently, often, always).

### 2.3. Data Collection

The sample was picked by an incidental sampling, in which similar percentages in the different variable demographics were sought at all times. The headmasters of the educational centers were contacted to seek collaboration by e-mail (with a cover letter and detailed information about the research attached). After obtaining permission, application of this tool was applied, specifying the objectives of the research. The answers were treated with total confidentiality and will always maintain an anonymous nature.

### 2.4. Data Analysis

Statistic software SPSS v. 23 and AMOS (IBM Corp.) were used for the analysis of the data. First, the psychometric properties of the study were assessed (item analysis, reliability, exploratory and confirmatory factorial analysis). Second, the percentages obtained globally and for each item were examined. Finally, an inferential analysis was developed through average scores and standard deviation, analysis of variance (ANOVA), and multiple a posteriori comparisons (Scheffé test). The size of the effect was also calculated (Cohen’s d).

## 3. Results

### 3.1. Psychometric Properties of Ordinary and Extraordinary Measures Survey for the Attention to Diversity.

First, an analysis of the 21 items was done on the survey related to ordinary and extraordinary measures for the attention to diversity. The average of the items fluctuated between 1.91 and 4.27, and in the standard deviation (SD) between 1.47 and 2.88. Likewise, it was proven that all the items fulfilled the norm from the asymmetry and kurtosis index values below two [18]. Afterwards, an exploratory factorial analysis was carried out by the extraction method of the main components and a posterior Varimax rotation. The adequacy index sample of Keiser–Meyer–Olkin (KMO) was 0.89 and the Bartlett Sphericity test was of significance (χ^2^ = 3235.731; gl = 210; *p* < 0.001), which indicated the factorial study’s feasibility. Following the Kaiser–Guttman rule, an initial solution of two factors with values over one was obtained, which explains 49.1% of the total variance on the attention-to-diversity measures. A factor grouped together the ordinary measure items and another the extraordinary measure items. The correlation between both dimensions of the survey reached a high level and was significant (r = 0.69, *p* < 0.01). All of the items showed communalities over 30% (Table 1).

Next, the factorial structure obtained was tested through a factorial analysis with the AMOS 20 version programmer [19] with an estimation method of maximum authenticity [20]. After testing this two-factor model, the analysis demonstrated an acceptable fit to the data, χ^2^ (169) = 853.366, *p* < 0.05, χ^2^/df = 5.050, Goodness of Fit Index –GFI- = 0.89, Adjusted Goodness of Fit Index –AGFI- = 0.91, and Root Mean Square Error of Approximation-RMSEA- = 0.05 [21,22].

Finally, the Cronbach alpha (α) index of Cronbach was calculated in order to estimate the internal consistency of the survey. Consequently, the survey showed a good internal coexistence in its totality (α = 0.80), as well as in each of its factors (ordinary measures: α = 0.82; extraordinary measures: α = 0.78) [23].

### 3.2. Knowledge and Use of Ordinary and Extraordinary Measures for the Attention to Diversity in CSE

The results showed a better knowledge by secondary education teachers with ordinary measures (72%) compared to extraordinary ones (60%). Additionally, after the adjusting of the frequency of use decoder in percentage with lower (never, rarely, seldom) or higher (frequently, often, always) use, the ordinary measures had a higher employ (51%) than the extraordinary ones (42%) (Table 2).

Likewise, ordinary measures which were more widely known by secondary education teachers were didactic planning adaptation to the environment and pupils (88.7%), educational reinforcement and support by teachers with free periods (88.3%), adaptation of the organizational structure of the center (timetables, groupings, spaces) and management of the classroom to the characteristics of the pupils (86.5%), adaptation of time and evaluation tools or the procedures of evaluation (85.8%), and reinforcement programmers in basic training areas (84.4%). The more highly known extraordinary measures were curricular adaptations (95.6%), support by a therapy pedagogue or speech therapist (92.9%), and curricular diversity programmers (83.4%). On the contrary, among the ordinary measures which were lesser known by teachers were curricular enrichment programmers (29.9%) and social ability programmers (45.1%), while with the extraordinary measures were language acquisition groups (31.2%) and curricular adaptation to competency groups (32.7%).

Finally, the ordinary measures more often used by secondary education teachers were didactic adaptation planning to the environment and pupils (70.3%), adaptation of the organizational planning organization of the school and classrooms to the characteristics of the pupils (65.1%), and the adaptation of times, tools, and assessment (62.8%). Likewise, among the more frequently used extraordinary measures we found the support of a therapy pedagogue and speech therapist (71.7%), curricular diversity programmers (66.6%), and curricular adaptations (65.6%).

### 3.3. The Significant Differences in Using the Ordinary and Extraordinary Measures

The results showed a greater use of the ordinary measures to the attention to diversity for secondary education pupils by male teachers who were between 45 and 50 years old, with a working time of less than 12 years and a bachelor’s degree, and who lived in provinces with a lesser number of inhabitants. In the same way, the teachers from the secondary schools that used the extraordinary measures with greater diligence were female, aged between 51 and 55 years old, with less than 12 years’ work experience, who held a doctorate degree, and who resided in provinces with a lesser number of inhabitants. To the contrary, the secondary school teachers with lesser use of the ordinary measures were females, older than 55 years old, with more than 18 years’ work experience, who held bachelor’s degrees, and who resided in provinces with a greater number of inhabitants. Likewise, a lesser use of the extraordinary measures were used by male teachers, younger than 45 years old, with work experience of over 18 years, possessing a bachelor’s degree, and living in provinces with a greater number of inhabitants (Table 3).

Next, the variance analysis showed significant differences in the work experience variable (F_2, 449_ = 358, *p* < 0.005, d = 0.46) for ordinary measures and in the variation of degrees (F_2, 449_ = 3.18, *p* < 0.001, d = 0.51) and province (F_3, 448_ = 3.09, *p* < 0.005, d = 0.48) for the extraordinary measures. A posteriori analysis (Scheffé test) showed significant differences (*p* ≤ 0.05) in the work experience variable, between those with less than 12 years of work experience (M = 46.27), those with more than 18 years (M = 40.45), and those who ranged from 12 to 18 years (M = 41.18 ); in the degree variable, there were differences between those with a doctorate degree (M = 30.52 ) and those with a certification (M = 25.47). Finally, in the province variable, there were differences between those who lived in small-population areas (M = 28.96) and those who resided in larger population areas (M = 25.58). Moreover, all the variables showed a half effect size (Cohen’s d between 0.46 and 0.50).

Therefore, the results expressed a greater use of ordinary measures in secondary education teachers with a less than 12 years’ work experience and extraordinary measures in teachers with a doctorate degree who teach in provinces with a lesser number of inhabitants.

## 4. Discussion

The educative success from the attention to diversity can create a new utopia out of the education system if we overcome the view focused on pupils with more difficulties and opt for an inclusive hypothesis based on education for everyone [1,3,12,24,25]. Teachers consider that schools are moving increasingly towards inclusive educational practices; however, more time is required to modify, integrate, and assume behaviors that are more prone to diversity. The main objectives of this study were to assess the psychometric properties of the survey on the ordinary and extraordinary measures of attention to diversity in Secondary School Education (CMOEAD), to check the knowledge and use of attention-to-diversity measures in Secondary Education Schools, and to analyze whether the demographic variables predict significant differences in the use of those measures. Thus, in general, the results obtained have shown that attention to diversity goes beyond the difficulties and specific educational needs of some students, and should seek more personalized attention within the group-class.

The results obtained show that the first starting hypothesis is confirmed (H_1_: “It is inferred that the CMOEAD questionnaire presents adequate psychometric properties for the evaluation of measures of attention to diversity”. The discoveries of this study showed appropriate characteristics of viability and validity of the survey, supporting its two-dimensional view (ordinary and extraordinary measures) on the population considered. Results suggested that the survey adequately captured the hypothesized dimensions for the attention-to-diversity measures and showed appropriate psychometric properties, which made it recommended for its use in the educational field.

With regard to the second hypothesis, “H_2_: It is predicted that there are discrepancies between knowledge and use in measures of attention to diverse students”, the data supported our proposition. Practices based on the segregation of the secondary school pupils were evident in this research: greater knowledge and use of specific measures for a single pupil or group of pupils (curricular adaptations, support from a pedagogue and speech therapist, educative reinforcement, planning adaptations or organizational structures) as opposed to the ones focused on the entire group-class (language acquisition groups or curricular adaptation, social abilities programmers). Additionally, a better knowledge and use of ordinary measures compared to extraordinary ones was manifest, following several studies which establish the continuation of the application that goes from the more general and less significant measures (ordinary) to the more significant and individual ones (extraordinary) [26,27]. Moreover, it was confirmed that the measures of attention to diversity that are most well-known are the ones more frequently used by teachers in this stage.

The research also showed that the attention to diversity measures most used by secondary education teachers were adaptations to didactic planning, organizational structures, time adaptations, tools or assessment procedures, pupils’ characteristics, support by the specialists, and planning adaptations. On the contrary, the measures of attention to diversity that showed a less frequent use were specific programmers (social abilities, enrichment, personalized) and language acquisition groups or adaptation to curricular competence. This corroborates the reductionist view that education centers keep maintaining with attention to diversity. This consideration was emitted by several authors [28,29,30].

Finally, our results partially supported the third working hypothesis, “H_3_: Socio-demographic variables are predicted to determine significant differences in the use of measures for the care of diverse students”, since the data showed significant statistical differences and found that the teachers more inclined to use ordinary and extraordinary measures for the attention to diverse pupils were female or male, with an age range between 45 and 55 years old, with little work experience (less than 12 years), with a doctorate or bachelor’s degree, and who taught in interior provinces (Lugo and Ourense). Likewise, the a posteriori analysis (Scheffé test) showed a higher use of measures to attend to diversity in teachers with less experience, a doctorate degree, and who developed their work in provinces with a low number of inhabitants. In that sense, it is important to highlight the importance of beliefs and attitudes that teachers had on diversity, which is not put into practice for the inclusive educational activities in Secondary Education Schools [31,32,33,34].

It is prudent to emphasize that the universe that was studied here is partial, so the results cannot be completely translatable to the rest of the population. The main limitation of this study was that the information gathered and analyzed was exclusively focused on perceptions and perspectives of CSE teachers, without contrasting this information against that from other actors such as parents, school counselors, and other professionals involved in the school community. Moreover, the study was restricted to quantitative methodology based on a self-report questionnaire, an instrument whose efficacy relies entirely on the participants’ truthfulness.

For that reason, in future research it would be important to complete the vision offered in this study with other studies that consider diversity attention from the point of view of other professionals (educative inspectors, specific counselling teams, school counselors, manager teams, therapeutic pedagogy specialists, and tutors).

## 5. Conclusions

In conclusion, attention to diversity can not only benefit the most vulnerable students, it should answer needs and have them correspond with potentials and possibilities, from participation and learning processes for all pupils. Thus, the study enables a new tool (CMOEAD survey), built from the knowledge of secondary education teachers, which can lead to a process of improvement in teaching-learning with a predominant focus on inclusion [8,28,35]. As a consequence, if the main educative challenge is to provide answers that guarantee the attention to diversity, it seems evident that it requires teachers, able to recognize and respond to pupils’ heterogeneity, who are trained, are prepared, and above all, have positive attitudes and take responsibility for turning their attention to diversity challenge in the educational system [32,36,37,38,39]. However, the opportunity for transformation and change in educational centers towards attention to diversity will not exist if quality education is not provided for all students [1,8,40].

## Figures and Tables

**Table 1:** Mean scores, SD, asymmetry, kurtosis, factorial relevance, homoscedasticity, correlation, and reliability of the items.

ITEM	MEAN	SD	ASYMMETRY	KURTOSIS	FACTORS		h2
					Ordinary Measures	Extraordinary Measures	
2	4.09	1.51	−0.63	−0.44	0.82		0.67
4	3.85	1.54	−0.45	−0.75	0.76		0.57
3	3.21	1.73	0.50	−1.32	0.75		0.58
1	3.91	1.54	−0.51	−0.66	0.71		0.52
11	2.53	1.74	0.65	−1.02	0.54		0.41
6	3.46	1.84	−0.14	−1.43	0.52		0.40
7	3.98	1.63	−0.48	−0.85	0.51		0.41
5	3.75	1.73	−0.36	−1.13	0.50		0.40
10	3.63	1.73	−0.25	−1.15	0.49		0.39
12	2.35	1.71	0.87	−0.68	0.49		0.38
8	1.99	1.57	1.31	0.29	0.39		0.37
9	4.13	2.88	1.01	1.29	0.35		0.35
21	1.96	1.55	1.33	0.35		0.68	0.51
18	2.61	1.99	0.64	−1.32		0.67	0.47
20	1.91	1.52	1.44	0.68		0.65	0.43
17	4.12	1.78	−0.59	−0.94		0.61	0.38
19	2.43	1.79	0.80	−0.87		0.48	0.37
13	4.14	1.47	−0.47	−0.70		0.47	0.40
15	4.27	1.63	−0.69	−0.63		0.43	0.35
14	3.23	1.87	0.07	−1.40		0.43	0.34
16	2.51	1.83	0.75	−0.96		0.39	0.34
% Variance explained					26.9%	22.2%	49.1%
Correlations between factors					0.69 (significant at level 0.001)		
Reliability (Cronbach alpha index)					α = 0.82	α = 0.78	α = 0.80

**Table 2:** Level of knowledge and frequency of use of ordinary and extraordinary measures for the attention to diversity of the CSE.

Attention to Diversity Measures in Secondary Education	% KNOWLEDGE		% USE	
	YES	NO	LOW	HIGH
**Ordinary Measures for Attention to Diversity**				
Adequacy in the structural organization of the school and classroom to pupil characteristics	86.5	13.5	34.9	65.1
Adequacy of the didactic planning for the environment and pupils	88.7	11.3	29.7	70.3
Methodology based on collaborative work in heterogeneous groups, tutoring among equals, learning through projects, and other activities that foster inclusion	73	27	54	46
Adapting the time and instruments or procedures of evaluation	85.8	14.2	37.2	62.8
Educative attention and coexistence social classes, and measures destined to improve coexistence	79.9	20.1	41.4	58.6
Group flexibility	72.6	27.4	45.3	54.7
Educational reinforcement and support by teachers with free periods	88.3	11.7	35	65
Curricular enrichment programs	29.9	70.1	79.96	20.1
Reinforcement programs in basic areas of learning	84.4	12.6	46.7	50.3
Recovery programs	79.4	20.6	53.2	56.8
Specific personalized programs	50	50	69.1	30.9
Social abilities programs	45.1	54.9	73.7	26.3
**Extraordinary Measures for Attention to Diversity**				
Curricular adaptations	95.6	4.4	34.4	65.6
Flexible groupings	65.7	34.3	52.7	47.3
Support from teachers specialized in therapy-pedagogy or speech therapists	92.9	7.1	28.3	71.7
Flexibility in the duration of schooling	48.5	51.5	70.6	29.4
Curricular diversity programs	83.4	16.6	33.4	66.6
Initial professional qualification programs/basic vocational training	45.4	54.6	65.7	34.3
Educational attention to pupils who, due to different circumstances, present difficulties for regular attendance at an educational center	47.6	52.4	70.8	29.2
Language-acquisition groups	31.2	68.8	82.1	17.9
Curricular adaptation to competency groups	32.7	67.3	80.9	19.1
TOTAL	Ordinary measures: 72% Extraordinary measures: 60%		Ordinary measures: 51% Extraordinary measures: 42%	

**Table 3:** Averages, SD, variance analysis, and size of the effect of ordinary and extraordinary measures, taking into account age, gender, work experience, degree, and province.

Variables	Ordinary Measures					Extraordinary Measures				
	*Mean*	*SD*	*F*	*p*	*d*	*Mean*	*SD*	*F*	*p*	*d*
Gender										
Woman	40.75	13.03	1.47	0.676	-	27.38	8.91	1.52	0.638	**-**
Men	41.27	12.39				26.96	9.57			
AGE										
Less than 45	41.02	12.02	1.52	0.780	-	27.06	9.17	1.43	0.890	-
Between 45 and 50	41.34	12.73				27.32	10.39			
Between 51 and 55	41.31	13.62				27.43	8.71			
Older than 55	39.92	13.80				27.26	8.36			
Work Experience										
Less than 12	46.27	11.77	3.58	0.002	0.461	28.65	10.95	1.69	0.502	-
Between 12 and 18	41.18	11.52				27.82	9.04			
Older than 18	40.45	13.15				26.93	9.05			
Degree										
Certification	39.91	12.70	1.59	0.743	-	25.47	8.56	3.18	0.000	0.508
Bachelor’s degree	41.16	12.86				27.40	9.11			
Doctorate degree	41.09	12.28				30.52	11.13			
Province										
Coruña	40.18	13.65	2.84	0.137	-	28.68	10.68	3.09	0.027	0.485
Lugo	42.02	11.96				28.96	6.09			
Ourense	42.01	12.23				26.28	8.79			
Pontevedra	37.79	12.95				25.58	7.74			
